# Adherence to RCPsych Standards for Physical Health Monitoring and Health Promotion in Patients Open to the North Wales Early Intervention Psychosis (EIP) Service

**DOI:** 10.1192/bjo.2024.345

**Published:** 2024-08-01

**Authors:** Zeenish Azhar, Javier Mendieta

**Affiliations:** Betsi Cadwaladr University Health Board, Wrexham, United Kingdom

## Abstract

**Aims:**

- The audit aims to improve the quality of physical health monitoring and physical health interventions that the EIP service provides to people with psychosis.
- To ensure adherence to RCPsych standards for physical health monitoring in patients with First Episode Psychosis.
- To ensure adherence to RCPsych standards for provision of required physical health interventions and health promotion in patients with First Episode Psychosis.

**Methods:**

- A retrospective case note audit and re-audit was conducted for 13 patients on the caseload of the North Wales EIP service from December 2022 to December 2023.
- The case notes were audited against RCPsych standards for physical health monitoring and physical health interventions using an adapted version of the National Clinical Audit of Psychosis (NCAP) audit tool.

**Results:**

- Alcohol and substance misuse screening status improved to 100% in re-audit.
- There was significant improvement noted in Hypertension, Body Mass Index and Cholesterol screening.
- Mental health medication review, advice or referral for diet and exercise with regards to weight gain/obesity and hypertension improved to 100%.
- No specialist interventions were offered around health promotion and illness prevention as most of the patients were either not in the abnormal range, identified as high risk for developing the above mentioned physical health conditions or refused to have interventions for these conditions.
- A definite increase was observed in frequency of interventions being reviewed and reoffered for those accepting and declining interventions at baseline.

**Conclusion:**

- Training for staff to complete bloods and physical health screening.
- Increase availability of equipment to carry out physical health screening.
- Monthly, three and six monthly prompts in the case notes for staff to discuss physical health interventions with patients.
- Staff to use headings for physical health screening and interventions to improve documentation in case notes.

